# Centromere sequence-independent but biased loading of subgenome-specific CENH3 variants in allopolyploid *Arabidopsis suecica*

**DOI:** 10.1007/s11103-024-01474-5

**Published:** 2024-06-14

**Authors:** Raheleh Karimi-Ashtiyani, Ali Mohammad Banaei-Moghaddam, Takayoshi Ishii, Oda Weiss, Jörg Fuchs, Veit Schubert, Andreas Houben

**Affiliations:** 1 Department of Biotechnology, Faculty of Agriculture, Tarbiat Modares University, Tehran, 1497713111 Iran; 2 Laboratory of Genomics and Epigenomics (LGE), Department of Biochemistry, Institute of Biochemistry and Biophysics (IBB), University of Tehran, Tehran, 1417614335 Iran; 3 Arid Land Research Center (ALRC), Tottori University, 1390 Hamasaka, Tottori, 680-0001 Japan; 4 Leibniz Institute of Plant Genetics and Crop Plant Research (IPK), Gatersleben, 06466 Seeland, Germany

**Keywords:** Allopolyploidization, CENH3, Centromere plasticity, Haploid, Species evolution, Uniparental chromosome elimination

## Abstract

Centromeric nucleosomes are determined by the replacement of the canonical histone H3 with the centromere-specific histone H3 (CENH3) variant. Little is known about the centromere organization in allopolyploid species where different subgenome-specific CENH3s and subgenome-specific centromeric sequences coexist. Here, we analyzed the transcription and centromeric localization of subgenome-specific CENH3 variants in the allopolyploid species *Arabidopsis suecica.* Synthetic *A. thaliana* x *A. arenosa* hybrids were generated and analyzed to mimic the early evolution of *A. suecica*. Our expression analyses indicated that CENH3 has generally higher expression levels in *A. arenosa* compared to *A. thaliana*, and this pattern persists in the hybrids. We also demonstrated that despite a different centromere DNA composition, the centromeres of both subgenomes incorporate CENH3 encoded by both subgenomes, but with a positive bias towards the *A. arenosa*-type CENH3. The intermingled arrangement of both CENH3 variants demonstrates centromere plasticity and may be an evolutionary adaption to handle more than one CENH3 variant in the process of allopolyploidization.

**Supplementary Information:**

The online version contains supplementary material available at 10.1007/s11103-024-01474-5.

## Introduction

Centromeres are required for the correct segregation of chromosomes. This chromosome domain is the assembly site for the proteinaceous kinetochore complex, which dictates the correct distribution of sister chromatids and their transmission to daughter cells during mitosis and meiosis. Therefore, it was expected that centromeric sequences should have conserved sequence characteristics to be identified by kinetochore proteins. However, it was shown that the function of most centromeres is epigenetically determined and largely independent of the underlying sequences (Talbert and Henikoff 2020). Centromeric nucleosomes are distinguished by the replacement of the canonical histone H3 with the centromere-specific histone H3 variant (CENH3, also called CENP-A). This is a mark for the active centromere, which epigenetically determines centromere function in most eukaryotes (Henikoff and Dalal 2005). Defects in the centromeric chromatin may lead to missegregating chromosomes, and result in aneuploidy, a frequently observed phenomenon in cancer (Tomonaga et al. 2003).

Besides some diploid plant species encoding multiple functional CENH3 paralogs, e.g. *Hordeum vulgare* (Sanei et al. 2011a), *Pisum sativum* (Neumann et al. 2012), *Mimulus* (Finseth et al. 2015) and cowpea, b), most diploid eukaryotes encode one variant of CENH3. *Drosophila virilis,* a diploid animal with two CENH3 variants (Cid1 and Cid5), revealed mutually exclusive gametic specialization of divergent CENH3 paralogs (Kursel et al. 2021).

In allopolyploid plants, in contrast, each parental subgenome might possess its own type of CENH3 operating in the context of multiple species-specific centromeric sequences. To analyze whether multiple CENH3 variants coexist in a hybrid genetic background, the expression of CENH3 variants was studied in some allopolyploids or artificial hybrid species. In allotetraploid *Oryza* species, both CENH3 variants are transcribed, and no *CENH3*-type preferential expression pattern was found (Hirsch et al. 2009; Li et al. 2010). In three different *Brassica* allotetraploid species, either co-transcription of both parental CENH3s or the suppression of one parental CENH3 variant was detected (Wang et al. 2011). On the other hand, uniparental silencing was revealed in an oat-maize chromosome addition line in which the maize-derived CENH3 was silenced (Jin et al. 2004). In oat x pearl millet hybrid, despite the biparental expression of both CENH3 genes, only the oat-type CENH3 was incorporated into the centromeres of both species in the hybrid embryo (Ishii et al. 2015a, b). In a study on the reconstructed wheat chromosome 1B with a hybrid wheat-rye centromere in the background of wheat, it was shown that only the rye-derived centromere part incorporates CENH3 of wheat (Karimi-Ashtiyani et al. 2021a). Further, uniparental centromere inactivation due to impaired CENH3 incorporation has been demonstrated during early embryogenesis in unstable hybrid of *Hordeum vulgare* x *H. bulbosum* (Sanei et al. 2011a). In contrast, cross-species incorporation of CENH3 variants was observed despite centromere-sequence differences in stable hybrids (Sanei et al. 2011a), emphasizing the importance of compatible parental centromeres in the process of speciation. Thus, different scenarios exist for how multiple CENH3 variants are handled in the hybrid background.

To study the dynamics of multiple CENH3s in hybrids between Arabidopsis species, we analyzed the subgenome-specific CENH3 variants in natural and synthetic allopolyploid *Arabidopsis suecica* as well as *A. thaliana* x *A. arenosa* F1 hybrids. *A. suecica*, a natural hybrid of *A. thaliana* and *A. arenosa,* which is estimated to have originated around 16,000 years ago (Novikova et al. 2017), is a valuable model for investigating the genomic and epigenomic changes associated with polyploidization (Comai et al. 2000). It offers the ability to replay evolution by producing synthetic hybrids, making it an excellent candidate for such studies. In the plant CENH3 pioneering study from Talbert et al. (2002), the *A. thaliana-*specific CENH3 antibody (also known as anti-HTR12) was generated, and immunostaining of natural and synthetic allopolyploid *A. suecica* chromosomes demonstrated that CENH3 from *A. thaliana* can be detected at all centromeres*.* However, it was not examined whether the same hybrids also included *A. arenosa*’*s* CENH3. Our analysis revealed that despite a different centromere DNA composition, the centromeres of the investigated allopolyploids incorporate CENH3s of both parental genomes. However, already after the formation of the F1 hybrid, the contribution of *A. arenosa*-derived CENH3 is higher than that of *A. thaliana* CENH3.

## Materials and methods

### Plant material and crossing procedure

*A. thaliana* (N3151, 2n = 4× = 20, ecotype Columbia-0 (Col-0)), *A. arenosa* (N3901, 2n = 4× = 32, ecotype Care-1), *A. suecica* synthetic allopolyploid hybrid N22665 and the natural hybrid Sue2 (2n = 4× = 26) were obtained from the European Arabidopsis Stock Centre (NASC). The synthetic allotetraploid *A. suecica* (N22665) had been produced by the crossing of autotetraploid *A. thaliana* (N3151) and autotetraploid *A. arenosa* (N3901) plants. For interspecific crossing to produce F1 hybrids, the closed buds of *A. thaliana* (N3151) were emasculated, and their stigmas were pollinated with *A. arenosa* (N3901) pollen. Plants were first grown under an 8 h photoperiod per day, 22 °C /18 °C day/night temperature. After 4 weeks, plants were transferred to long-day conditions (16 h photoperiod per day).

### Genomic DNA and RNA extraction, PCR and quantitative PCR

Genomic DNA was extracted from leaf tissue using a DNAeasy plant mini kit (Qiagen). Total RNA was isolated using the Trizol method (Chomczynski and Sacchi 1987). The absence of DNA contamination in RNA was confirmed by PCR using *ELF1*-specific primers (Suppl. Table 1). The cDNA was synthesized with 1 µg of DNase-treated total RNA using a RevertAid H Minus first-strand cDNA synthesis kit using oligo dT primers (Fermentas). Primers specific for the constitutively expressed *Actin 2* gene (At3g18780; Suppl. Table 1) were used as a control for an equal amount of gDNA and cDNA as well as for calibration in quantitative comparisons. The relative transcript level of *A. thaliana*-and *A. arenosa*-originated-*CENH3* was measured by qPCR using species-specific primers (Suppl. Table 1). 10 μl of PCR mixture contained 1 μl of cDNA template, 5 μl of 2× Power SYBR Green PCR Master Mix (Applied Biosystems), 0.33 mM of the forward and reverse primers for each gene. Reactions were run in an Applied Biosystems 7900HT Fast Real-Time PCR System, and data were analyzed with SDS software v2.2.2. The quantitative PCR was performed using the following conditions: 95 °C for 10 min, followed by 40 cycles at 95 °C for 15 s and an annealing temperature of 60 °C for 60 s. The specificity and efficiency of both primers were determined by qPCR using a dilution series of plasmids of cloned full-length cDNA of *A. thaliana* and *A. arenosa CENH3* genes. A similar *Ct* value (the PCR cycle at which the fluorescent signal of reporter dye exceeds background level) for an equal amount of plasmid and absence of amplification with the plasmid of the opposite CENH3 variant indicated that both primer pairs can amplify specific transcripts with the same efficiency.

### Genome size determination by flow cytometry and flow sorting of nuclei

The genome size of putative hybrid plants was estimated using *Raphanus sativus* ‘Voran’ (1.11 pg/2C, Gatersleben gene bank accession number: RA 34) as an internal reference standard in comparison to the values obtained for their tetraploid parents. For this, roughly 0.5 cm^2^ of fresh leaf tissue was chopped together with the internal reference standard in a Petri dish with a sharp razorblade in nuclei isolation buffer (Galbraith et al. 1983) supplemented with propidium iodide (50 µg/ml) and DNase-free RNase (50 µg/ml). The resulting nuclei suspension was filtered through a 50 µm CellTrics filter (Sysmex-Partec) and measured using a FACStar^PLUS^ cell sorter (BD Biosciences). The means of the nuclear peaks were determined using the software CellQuest (BD Biosciences) and the DNA contents calculated as described (Dolezel et al. 2007). For the parental tetraploid species *A. thaliana* (N3151) and *A. arenosa,* we performed 12 and six measurements, respectively. For the putative hybrid plants between one and six independent measurements were done.

For sorting of nuclei, leaf tissue was fixed in 4% formaldehyde, washed in Tris buffer, chopped in nuclei isolation buffer LB01 (Dolezel et al. 2007) and stained with 4′,6-diamidino-2-phenylindole (DAPI, 1 µg/ml final concentration) as described in (Ahmadli et al. 2023). The sorting was performed on a BD Influx cell sorter (BD Biosciences) equipped with a 355 nm UV laser using the BD FACS Sortware software. Afterwards, the sorted nuclei suspension was mixed with equal amounts of sucrose buffer on a microscopic slide and dried overnight (for details, see (Ahmadli et al. 2023)). Slides were either directly used for immunostaining or transferred for longer storage to - 20 °C.

### Generation of an *A. arenosa*-specific CENH3 antibody

The peptide RTKHFATKSRTGNRTDN was used to generate an *A. arenosa* CENH3-specific (anti-AaCENH3) polyclonal antibody (Suppl. Figure 1a). Peptide synthesis, immunization of guinea pigs, and peptide affinity purification of antisera were performed by Pineda, Antibody-Service (Berlin, Germany).

### Fluorescence in situ hybridization, indirect immunostaining and microscopy

*A. thaliana* (AtCEN) (Matsushita et al. 2012) and *A. arenosa* (AaCEN ()) centromere-specific FISH probes were generated by PCR using the primer pairs AtCEN-F/R and AaCEN-F/R, respectively (Suppl. Table 1). PCR was performed with *Taq* DNA polymerase with a block preheated to 94 °C and an initial denaturation of 94 °C for 2 min followed by 32 cycles of 94 °C for 25 s, 57 °C for 30 s, and 72 °C for 40 s. The probes were labeled with FITC or Cy3 by nick translation.

The rabbit HTR12-specific antibody (Abcam, ab72001, (Talbert et al. 2002)) was used for the detection of *A. thaliana* CENH3 (anti-AtCENH3). Leaves and flower buds were fixed in 1× phosphate buffered saline (PBS) containing 4% paraformaldehyde (PFA) for 20 min at room temperature and then immediately washed in 1× PBS twice for 5 min each. Indirect immunostaining and fluorescence in situ hybridization were carried out as described by (Gernand et al. 2003) and (Ma et al. 2010), respectively. Imaging was performed by using an Olympus BX61 microscope and an ORCA-ER CCD camera (Hamamatsu). All images were collected in greyscale and pseudocoloured with Adobe Photoshop 6 (Adobe). Maximum intensity projections were done with the Analysis (Soft Imaging System) program. To achieve an optical resolution of ca. 120 nm, applying a 488 nm excitation, we performed spatial structured illumination microscopy (3D-SIM) using a 63x/1.40 objective of an Elyra PS.1 super-resolution microscope system and the software ZENBlack (Carl Zeiss GmbH). Image stacks were captured separately for each fluorochrome using 561, 488, and 405 nm laser lines for excitation and appropriate emission filters (Weisshart et al. 2016; Kubalova et al. 2021). The 3D-image stacks were used to generate Suppl. Movie 1 using the Imaris 9.7 (Bitplane) software.

### Classification of immunosignal patterns

To compare the immunostaining patterns of the subgenome-specific CENH3 variants, double immunostaining was performed using rabbit anti-AtCENH3 (red signals) and guinea pig anti-AaCENH3 (green signals) specific antibodies. For this we prepared slides from flow-sorted 2C and 4C leaf nuclei (the two dominating (endopoly)ploidy levels in young leaf tissue) to minimize background signals. In subsequent quantitative immunolabeling experiments, as there were no significant differences observed between 2C and 4C sorted immunolabeled nuclei, we proceeded to analyze them collectively. The immunosignals were classified based on their strength (strong or weak) and absence. The absence of signals was called “0”, strong AtCENH3 or AaCENH3 signals were called “AT” or “AA”, subsequently. Weak AtCENH3 or AaCENH3 signals were called “at” or “aa”, respectively. Therefore, nuclei with unbiased subgenome-specific CENH3 signals were called either ‘AT/AA’ or ‘at/aa’. Nuclei with biased CENH3 signal intensities were called “AT/aa”, “at/AA”, “AT/0”, “at/0”, “AA/0” or “aa/0”. For each genotype, at least 155 immunostained nuclei were characterized (Suppl. Table 2).

## Results

### Biased incorporation of species-specific CENH3 variants in allopolyploid *Arabidopsis suecica*

**Fig. 1:**
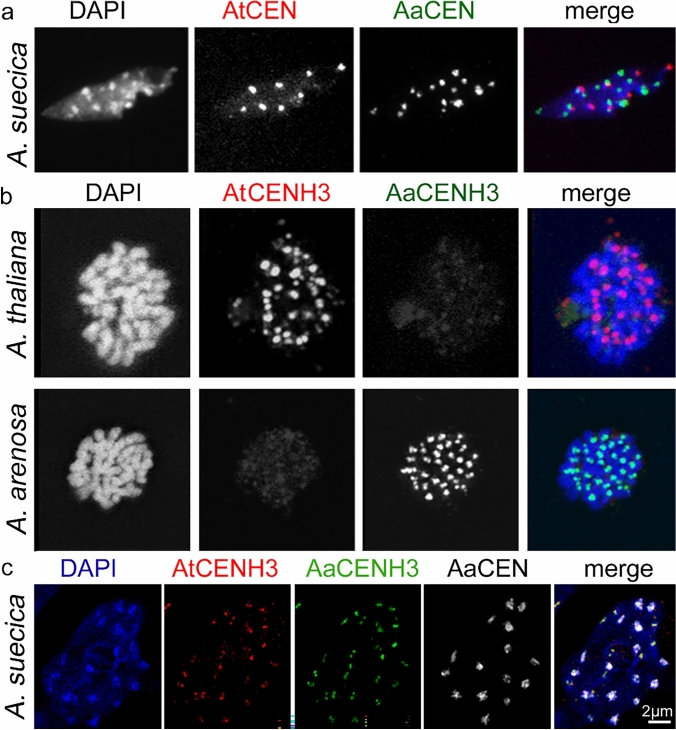
Verification of the hybrid nature of *A. suecica* and specificity of species-specific CENH3 antibodies. **a** The allopolyploid nature of *A. suecica*, confirmed by FISH on sorted 2C nuclei of *A. suecica* using a combination of *A. thaliana* (AtCEN) and *A. arenosa* (AaCEN) centromere-specific DNA probes. **b** Specificity of *A. arenosa* AaCENH3) and *A. thaliana* (AtCENH3) CENH3-specific antibodies confirmed by indirect immunostaining on mitotic chromosomes of *A. arenosa* and *A. thaliana*. **c** FISH with an *A. arenosa* centromere-specific probe after immunostaining confirmed that both CENH3 variants are centromere incorporated irrespectively of the underlying centromere sequence

To address whether subgenome-specific CENH3 variants undergo subgenome-specific centromere loading in an allopolyploid species, we used the allotetraploid *A. suecica* as a model. First, the hybrid nature of *A. suecica* was confirmed by multicolour FISH using centromere-specific DNA probes for *A. thaliana* (AtCEN) and *A. arenosa* (AaCEN) centromeres (Fig. 1a). After FISH, chromocenters revealed either *A. thaliana*- or *A. arenosa*-specific signals. Next, an *A. arenosa* CENH3-specific antibody (anti-AaCENH3) was produced and tested for specificity with mitotic chromosomes and interphase nuclei of *A. arenosa* (Fig. 1b, Suppl. Figure 1b). The absence of immunosignals in *A. thaliana* nuclei confirmed the species specificity of anti-AaCENH3. The species specificity of anti-*A. thaliana* CENH3 (anti-AtCENH3) was demonstrated as only the centromeres of *A. thaliana* but not of *A. arenosa* displayed immunosignals (Fig. 1b). Double immunostaining using both antibodies and subsequent FISH with the *A. arenosa* centromere-specific probe confirmed that both CENH3 variants are incorporated into all centromeres of *A. suecica* independent of their origin and sequence (Fig. 1c).

**Fig. 2:**
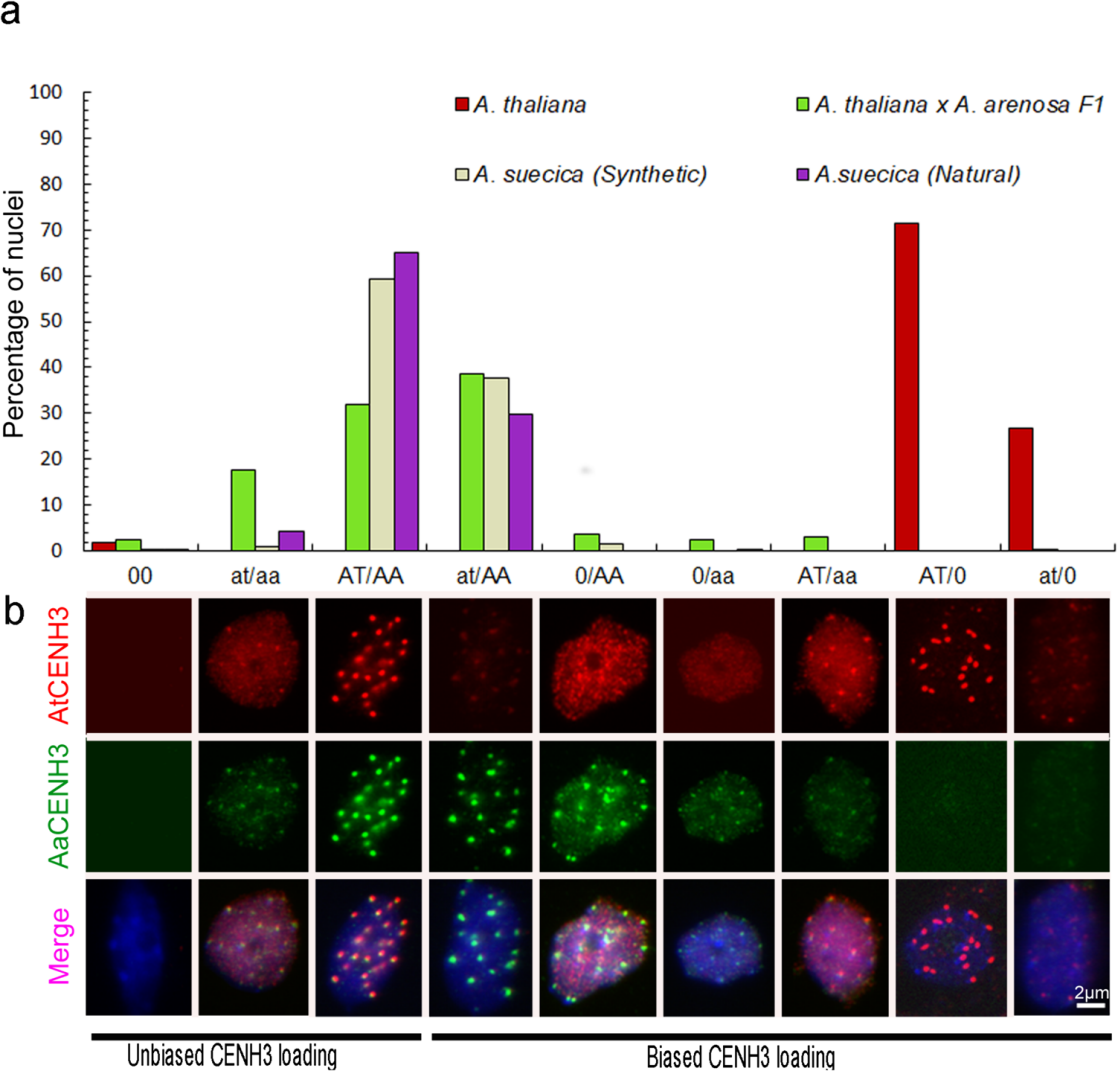
Sequence-independent but biased loading of parental CENH3 variants in *A. thaliana x A. arenosa* F1, synthetic and natural *A. suecica* plants. **a** Frequencies of distribution patterns of subgenome-specific CENH3 immunosignals on sorted nuclei. **b** Typical examples of labelled nuclei with different CENH3 distribution patterns. The immunosignals were classified based on their strength (strong or weak) and absence. The absence of signals was named “0”, strong AtCENH3 or AaCENH3 signals were named “AT” or “AA”, subsequently. Weak AtCENH3 or AaCENH3 signals were called “at” or “aa”, respectively. Therefore, nuclei with unbiased subgenome-specific CENH3 signals were named either ‘AT/AA’ or ‘at/aa’. Nuclei with biased CENH3 signal intensities were named “AT/aa”, “at/AA”, “AT/0”, “at/0”, “AA/0” or “aa/0”

Next, the centromeric localization of both subgenome-specific CENH3 variants was tested in synthetic allotetraploid *A. suecica* (N22665) plants. Double immunostaining revealed that about 60% of analyzed sorted nuclei incorporated equally both subgenome-specific CENH3s, resulting in either strong (AT/AA) or weak (at/aa) signals indicating an unbiased loading of subgenome-specific CENH3s (Fig. 2). In cases with a biased subgenome-specific CENH3 loading (i.e. AT/aa, at/AA, AT/0, at/0, 0/AA or 0/aa), we never found nuclei with strong AtCENH3 signals (i.e. AT/aa and AT/0) (Fig. 2 and Suppl. Table 2). In contrast, about 40% of nuclei, showed strong AaCENH3 signals (i.e. at/AA and 0/AA), indicating a biased loading of *A. arenosa* subgenome-specific CENH3.

To check whether the same bias exists in natural allopolyploid *A. suecica*, derived from ancient *A. thaliana* x *A. arenosa* hybridization events, we performed double immunostaining on sorted nuclei of *A. suecica* accession “Sue2”. About 70% of analyzed nuclei, had equally incorporated subgenome-specific CENH3s (Fig. 2). In cases with a biased subgenome-specific CENH3 loading (i.e. AT/aa, at/AA, AT/0, at/0, 0/AA or 0/aa), no nuclei with strong AtCENH3 signals (i.e. AT/aa and AT/0) were observed (Fig. 2 and Suppl. Table 2). However, about 30% of all nuclei, showed strong AaCENH3 signals (i.e. at/AA and 0/AA), demonstrating a similar biased *A. arenosa* CENH3 incorporation. To exclude that the observed signal differences of both species-specific CENH3 antibodies were not caused by a lower affinity of the AtCENH3 antibody, an additional control experiment was performed with tetraploid *A. thaliana*, the parent genotype we used for wide hybridization with *A. arenosa*. The same combination of antibodies resulted in nuclei with about 71% strong (AT/0) and 27% weak (at/0) AtCENH3-specific signals, respectively (Fig. 2). Thus, our findings indicate that both subgenome-specific CENH3 variants are incorporated into the centromeres of *A. suecica*, but with a positive bias towards *A. arenosa* CENH3.

### Preferential expression and loading of AaCENH3 become established immediately after hybrid formation

Crossing of both species was performed to determine whether the preferential *A. arenosa*-type CENH3 incorporation is already established in the first generation after the wide hybridization. Only pollination of tetraploid *A. thaliana* with tetraploid *A. arenosa* resulted in fertile seeds. As aneuploidy and chromosome elimination were described for crosses between *A. thaliana* and *A. arenosa* (Comai et al. 2000; Wright et al. 2009), flow cytometry was employed to identify successful hybridization events. In an initial pre-screening without an internal reference standard, one (plant 6) out of 30 plants was identified as diploid (Suppl. Figure 2a), while all other plants were confirmed to be tetraploid. Immunostaining with both types of CENH3 antibodies on nuclei of plant 6, confirmed the absence of the pollinator genome, as only up to 10 AtCENH3-specific signal clusters were found (Suppl. Figure 2b). Hence, here all *A. arenosa*-derived chromosomes were eliminated, likely during hybrid embryogenesis. Uniparental elimination of chromosomes is a common phenomenon in hybrids derived from distantly related species (reviewed in (Ishii et al. 2016). Furthermore, we noticed in the flow cytometric pre-screen an obvious variation in the peak positions between the measurements of the individual plants (Suppl. Figure 2c). Therefore, we determined the genome size of 24 putative F1 hybrids using *Raphanus sativus* as an internal reference standard and compared the data to values obtained for the tetraploid parents (Suppl. Figure 3a). For tetraploid *A. thaliana* and *A. arenosa,* we estimated genome sizes of 0.683 pg/2C and 0.825 pg/2C, respectively, indicating an expected genome size for the interspecific hybrid of 0.754 pg/2C. Among the analyzed plants, 13 plants revealed a genome size deviating by less than 3% from the expected value of a hybrid plant and were considered as euploid hybrid plants. 11 plants showed a deviation from the expected genome size of more than 3%, presumably as a result of aneuploidy. One plant is most likely the product of an *A. thaliana* selfing event or spontaneous doubling of haploid progeny (plant No. 25, Suppl. Figure 3a, b).

**Fig. 3:**
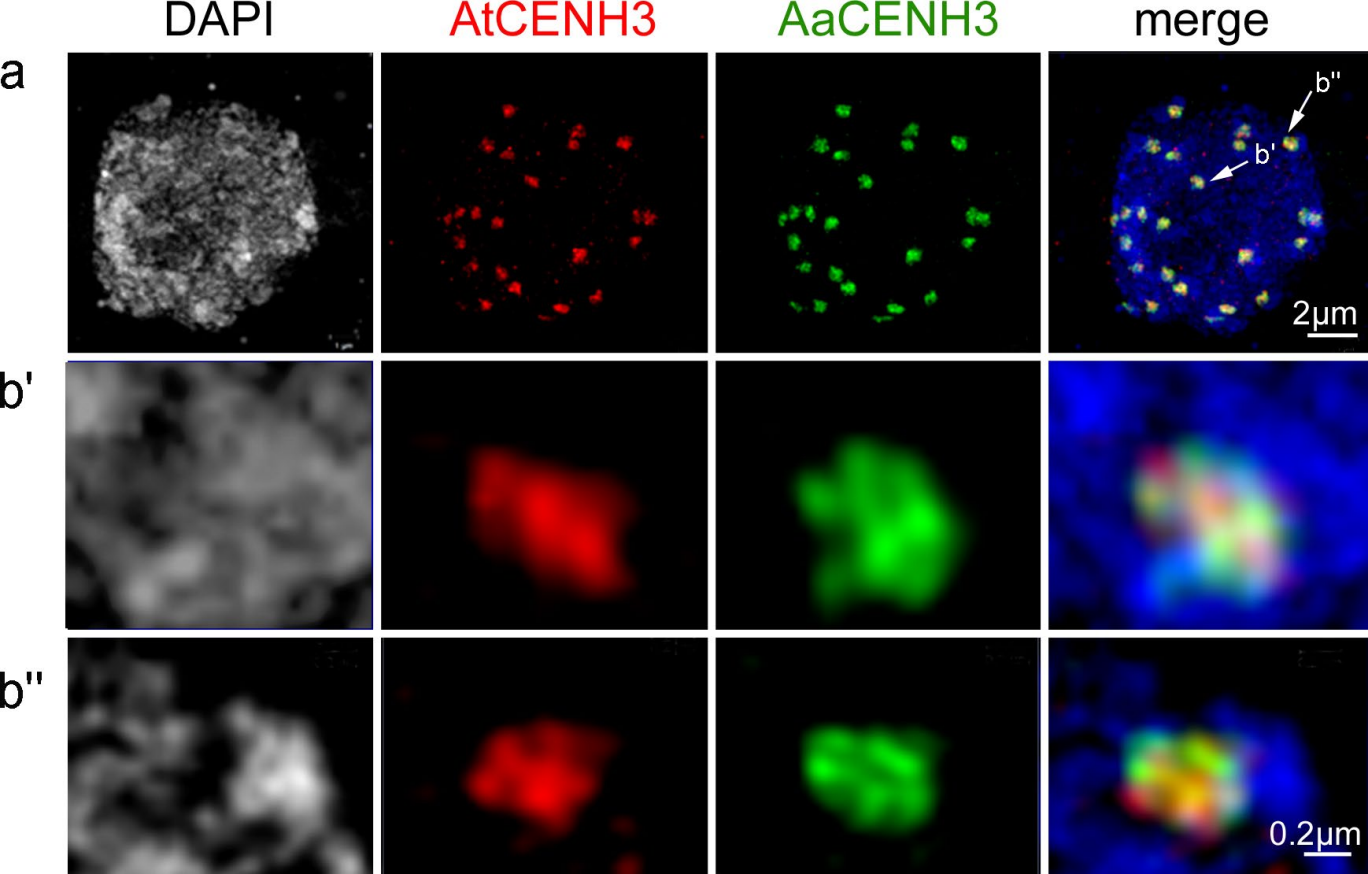
*A. thaliana* and *A. arenosa* CENH3s are loaded into neighbouring nucleosome arrays of *A. arenosa* x *A. thaliana* F1 centromeres. **a** Interphase distribution of anti-AtCENH3 (in red) and anti-AaCENH3 (in green) signals analyzed by super-resolution microscopy (3D-SIM) (see also Suppl. Movie 1). **b'**, **b''** Further enlarged centromeres

Based on the determined DNA contents, leaf nuclei of selected 3-month-old F1 hybrid plants were sorted on slides and analyzed by double immunostaining. About 50% of nuclei showed an unbiased loading of subgenome-specific CENH3 variants (AT/AA and at/aa nuclei). Out of the analyzed nuclei, only 3% showed stronger AtCENH3 signals (i.e. AT/aa, AT/0 and at/0 nuclei). In contrast, about 45% of CENH3-loaded nuclei, showed stronger *A. arenosa* than *A. thaliana* signals (i.e. at/AA, 0/AA and 0/aa nuclei) (Fig. 2, Suppl. Table 2), indicating a biased loading of *A. arenosa*-specific CENH3. The observed increase in the frequency of nuclei with an equal proportion of parental CENH3s (i.e., AT/AA and at/aa nuclei) from F1 to natural hybrids of *A. suecica* through generations suggests a gradual step-wise adaptation of CENH3 variant loading in allopolyploid Arabidopsis (Fig. 2, Suppl. Figure 4). Super-resolution microscopy confirmed the mixed composition of both parental CENH3s in F1 hybrid nuclei (Fig. 3, Suppl. Movie 1). However, the ultrastructure of AtCENH3 and AaCENH3 signals differed but intermingled, suggesting that the subgenome-specific CENH3 variants are preferentially loaded into different centromeric nucleosome arrays.

**Fig. 4:**
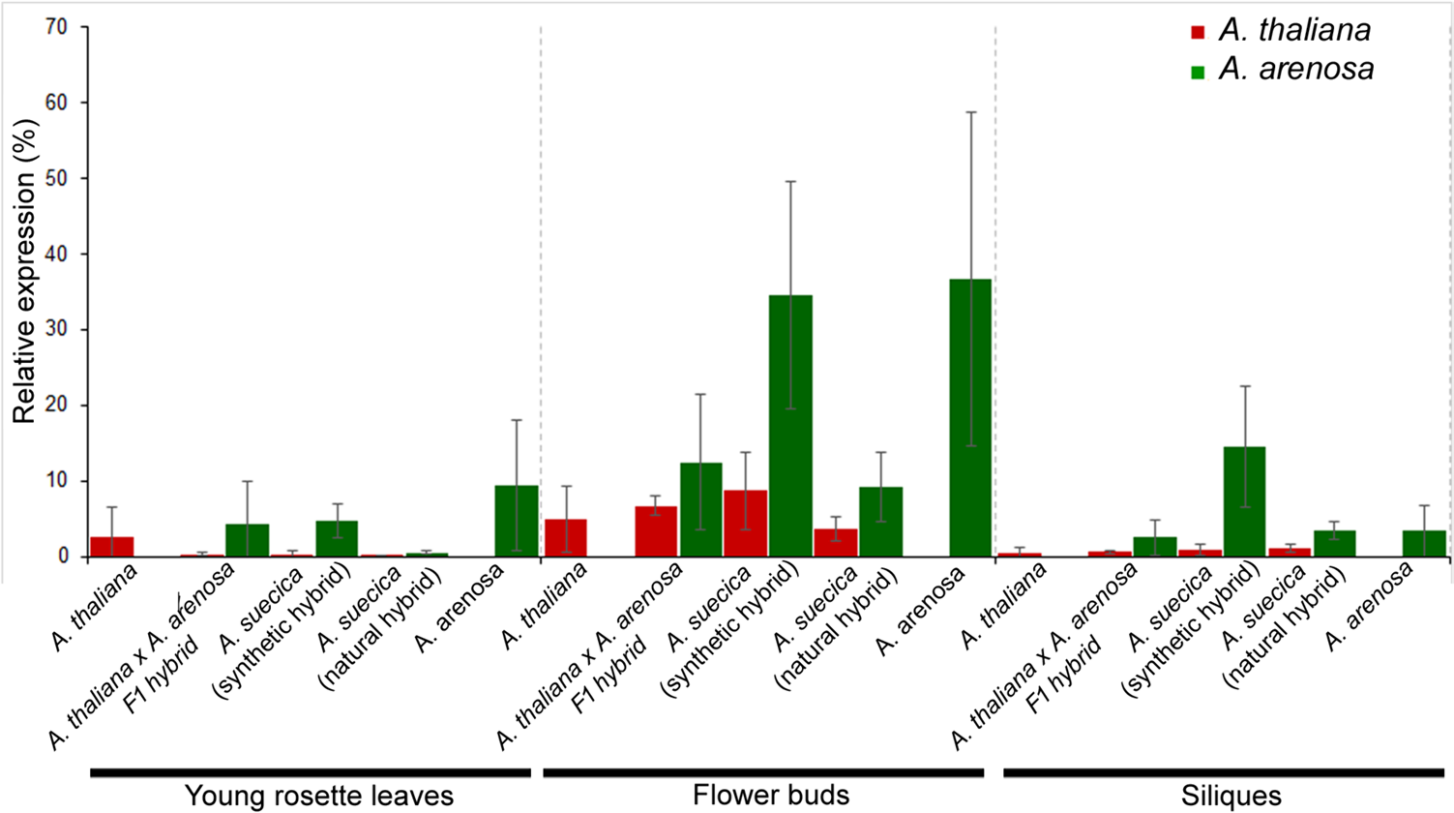
Expression analysis of *CENH3s* in *A. thaliana x A. arenosa* F1, synthetic and natural *A. suecica* plants*.* The expression levels of *CENH3* genes originating from *A. thaliana* and *A. arenosa* were investigated in parents and their hybrids using parent-specific-*CENH3* primers in different tissues of young rosette leaves, flower buds and siliques. The relative transcript levels of *CENH3* were quantified and normalized to *ACTIN2* (At3g18780). The relative magnitude of transcription was calculated by the following formula: R = 2^^(CtCENH3–CtActin2)^ (Livak and Schmittgen, 2001), where R = relative expression level. Bars represent the means of the relative transcript level of *CENH3* compared to *Actin2*. Error bars represent the standard deviation between three biological replicates

The observed biased CENH3 incorporation prompted us to analyze the transcription of both parental *CENH3s*. The relative transcript levels of *A. thaliana-* and *A. arenosa*-derived *CENH3* were quantified and then normalized to *ACTIN2* (At3g18780) after qPCR with *AaCENH3- *and *AtCENH3*-specific primer pairs (Fig. 4, Suppl. Table 1). In all analyzed tissues (young rosette leaves, flower buds, and siliques) of allopolyploid *A. suecica* (natural hybrid) and synthetic hybrids (F1, older synthetic hybrid), the relative expression of *A. arenosa CENH3* was higher in comparison to *A. thaliana*.

**Fig. 5:**
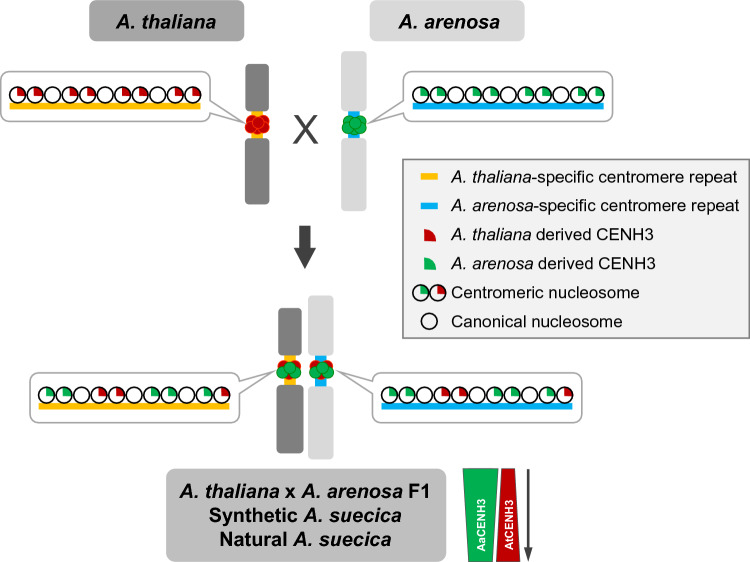
Model of the centromere sequence-independent but biased centromere loading of subgenome-specific CENH3s in allopolyploid *A. suecica.* The majority of centromeres of *A. thaliana* x *A. arenosa* F1 hybrid, synthetic and natural *A. suecica* incorporate CENH3s of both parental genomes, despite a different DNA centromere composition. In nuclei with biased loading of subgenome-specific CENH3, the *A. arenosa*-derived CENH3 is dominant. After several generations, the frequency of biasedly loaded subgenome-specific CENH3 nuclei decreases, and both subgenome-specific CENH3s load more balanced to the centromeres of natural *A. suecica* plants

In summary, we conclude that the majority (above 90%) of the centromeres of *A. thaliana* x *A. arenosa* F1 hybrid, synthetic and natural *A. suecica* incorporate CENH3s of both parental genomes, despite a different centromere DNA composition (Fig. 5). However, after the formation of the F1 hybrid, the contribution of *A. arenosa*-derived CENH3 increases compared to that of AtCENH3.

## Discussion

An invaluable plant model for examining the genomic and epigenomic alterations linked to polyploidization is *A. suecica,* a naturally occurring hybrid of *A. thaliana* and *A. arenosa* (Comai et al. 2000). It is a great option for these kinds of studies since it provides a means of simulating evolution through the creation of artificial hybrids. Here, we investigated whether distinct CENH3s, which originated from both parental species, incorporate centromere-sequence independently in a hybrid situation. Therefore, we used varying evolutionary ages of *A. suecica*, ranging from F1 hybrids produced by wide crossing of tetraploid *A. thaliana* and *A. arenosa* to resynthesize the species, a synthetic hybrid and natural *A. suecica*. We found that both CENH3s encoded by either subgenome incorporate into the centromeres of hybrid genotypes, even though the centromeric satellite repeats of both subgenomes share only 58 to 80% sequence identity (Kamm et al. 1995). This observation aligns with the findings of Talbert et al. (2002), who showed that anti-CENH3 of *A. thaliana* recognizes all centromeres in both synthetic and natural allopolyploid *A. suecica*. Also, a GFP::*A. arenosa* CENH3 construct can functionally replace CENH3 of an *A. thaliana* null mutant (*cenh3-1)* (Ravi et al. 2010). Analysis of alien CENH3s localization in *A. thaliana*- and tobacco-tissue cultured cells showed that in contrast to the CENH3 of rice, CENH3 of *A. thaliana* and tobacco incorporated into the centromeres of both tobacco and *A. thaliana* while CENH3 of the holocentric *Luzula nivea* became integrated only partially into the centromeres of *A. thaliana* cultured cells, suggesting that only evolutionally close CENH3s can target centromeres in alien species (Nagaki et al. 2010). In addition, the replacement of endogenous CENH3 in *A. thaliana* with CENH3 from related species demonstrated that CENH3 of heterologous species must be related to the *A. thaliana* CENH3 for functional complementation (Ravi et al. 2010; Maheshwari et al. 2017). Our observation that *A. arenosa*–derived CENH3 could localize to the centromeric satellite repeats of *A. thaliana* and *vice versa* is in agreement with these findings and confirm the close evolutionary relationship of both parent CENH3s. The coexistence of nuclei with different CENH3 patterns is likely caused by the multi-cell-type composition of leaves which were used for our analysis. Likely, the centromere composition is dynamically organized according to the needs of individual cell and tissue-types during development. A tissue-specific CENH3-type composition was also found in other species, like e.g. barley, cowpea and *D. virilis,* (Ishii et al. 2015a, 2020b; Kursel et al. 2021).

Our identification of a dihaploid *A. thaliana* plant after outcrossing tetraploid *A. thaliana* with tetraploid *A. arenosa* suggests that, like observed in other wide crosses, uniparental chromosome elimination could occur during embryogenesis. To our knowledge, we present the first evidence of subgenome elimination in an Arabidopsis interspecies hybrid by flow cytometry and immunostaining*.* However, to establish the best haploidisation conditions based on interspecific Arabidopsis hybridisation, future studies should follow.

Super-resolution microscopy revealed that AtCENH3 and AaCENH3 signals have an almost similar but not identical distribution in the centromeres of the hybrid plants, suggesting that both CENH3s do not exist as monomers in the same nucleosome. Instead, subgenome-specific CENH3s are loaded into separate nucleosome arrays to form distinct centromeric substructures. This phenomenon of CENH3 variant-specific distribution has also been observed in other plants, such as barley, a wheat 1BL/1RS translocation line, and cowpea, where different CENH3 variants occupy distinct centromeric nucleosomes (Ishii et al. 2015a, b; Yuan et al. 2015; Karimi-Ashtiyani et al. 2021a). The intermingled arrangement of different CENH3 variants in a centromere may be an evolutionary adaption to handle more than one CENH3 variant.

In a study focusing on the process of polyploidization, Burns et al. (2021) found that the speciation process in *A. suecica* was a gradual and adaptive evolutionary process rather than a sudden or drastic event. This gradual evolution was evidenced by the absence of massive changes in the genome arrangement and mobilization of transposable elements, as well as the lack of subgenome dominance in gene expression (Burns et al. 2021). This finding is consistent with the observed increased frequency of unbiasedly loaded nuclei of parental CENH3s from F1 to natural hybrids of *A. suecica* through generations in our study (Fig. 2, Suppl Fig. 4).

Also, flower bud chromosomes of an F1 hybrid plant and synthetic *A. suecica* revealed in all centromeres immunosignals of both CENH3 variants (Suppl. Figure 5a, b). However, the sample size was too small to conclude a biased occupancy of species-specific CENH3s in flower buds. Nevertheless, we cannot rule out the possibility that other *A. suecica* genotypes exhibit different CENH3 patterns, given that natural *A. suecica* is a product of several founding individuals rather than a single origin (Novikova et al. 2017).

Our expression analysis revealed that the dynamic of *CENH3* expression is similar in parents and their allopolyploid hybrids, with higher expression levels in tissues with rapidly dividing cells. Furthermore, our results indicate that *CENH3* has generally higher expression levels in *A. arenosa* compared to *A. thaliana*, and this pattern persists in the hybrids. This result is in line with a genome-wide expression study of Arabidopsis synthetic allotetraploids, which showed that most nonadditively expressed genes were predominantly repressed. This repression was particularly evident in genes that were expressed at higher levels in *A. thaliana* compared to *A. arenosa*, indicating a bias towards the *A. arenosa* genome (Wang et al. 2006). DNA hypomethylation of the *A. arenosa* subgenome in resynthesized and natural *A. suecica* allotetraploids may contribute to the observed upregulation of many genes involved in reproduction and adaptation (Jiang et al. 2021).

In conclusion, our analysis of transcription and centromeric localization of subgenome-specific CENH3 variants in the allopolyploid species *A. suecica* demonstrates that both parental CENH3 variants are retained, with a gradual increase of equal loading of subgenome-specific CENH3s during the progression from F1 to natural hybrids. This suggests the potential impact of centromere plasticity on establishing stable centromeres, genome integrity and evolution across generations in allopolyploid speciation.

### Supplementary Information

Below is the link to the electronic supplementary material.

Supplementary file1 (TIF 6281 KB) Generation and characterization of anti-AaCENH3 and anti-AtCENH3 antibodies. (a) Alignment of *A. thaliana* and A. arenosa *A. arenosa *CENH3 protein sequences. The polymorphic region (underlined, RTKHFATKSRTGNRTDN) was used to synthesize a peptide for generating AaCENH3-specific antibodies. Immunostaining of A. arenosa and A. thaliana*A. thaliana* nuclei with (b) anti-AtCENH3 and (c) anti-AaCENH3 antibodies demonstrate species-specific CENH3 recognition

Supplementary file2 (PPTX 281 KB) Flow cytometric analysis of the progeny of *A. thaliana *(4x) x A. arenosa*A. arenosa* (4x) crosses revealed in one plant elimination of all A. arenosa chromosomes. (a) Overlay of independent measurements of A. thaliana*A. thaliana* (4x) (red) and the putative hybrid plants 6 (grey) indicating its diploid status. (b) Double immunostaining on sorted nuclei of the 2x progeny (plant No. 6) using AtCENH3 (red) and AaCENH3 (green) antibodies. Only AtCENH3-specific and no AaCENH3 signals were found in nuclei, confirming the loss of A. arenosa*A. arenosa* chromosomes in the F1 plant. Bar represents 10 µm. (c) Overlay histogram of flow cytometric measurements of *A. thaliana* (4x) (red), A. arenosa *A. arenosa *(4x) (blue) and all tested putative F1 hybrid plants (grey) except plant 6 indicates severe differences in genome size between individual hybrid plants

Supplementary file3 (PPTX 1104 KB) Validation of the hybrid nature of *A. thaliana x A. arenosa* F1 plants. (a) The DNA content of F1 hybrid plants from crosses between A. thaliana *A. thaliana*and *A. arenosa *was determined by flow cytometry. Plants 25 and 27 were quite similar to Col-0, most likely due to failure in cross-pollination and consequently produced by self-pollination. The yellowish area represents the +/- 3% interval around the expected DNA content (0.754 pg/2C) of the hybrid plants based on measurements of both parental genome. Plants inside this interval were considered as euploid F1 hybrids (b) Phenotypes of some analyzed plants by flow cytometry. The images show the haploid plant 6 and the tetraploid *A. thaliana*(No. 25), along with F1 hybrids of 11, 16, and 24. The upper and lower panels represent side and top views, respectively. Note the differences in phenotypes

Supplementary file4 (PPTX 38 KB) The frequency of unbiased and biased CENH3 loaded nuclei A. thaliana x A. arenosa*A. thaliana x A. arenosa* F1plants, as well as synthetic and natural *A. suecica*. The percentage of unbiased (i.e., AT/AA and at/aa nuclei) and biased (i.e., AT/aa, at/AA, AT/0, at/0, 0/AA, or 0/aa nuclei) subgenome-specific CENH3 loaded nuclei is represented as a proportion of the total number of nuclei (sum of 2C and 4C) in each genotype

Supplementary file5 (TIF 528 KB) Coexistence of species-specific CENH3 variants in the centromeres of flower bud cells in F1 hybrid and synthetic A. suecica. *A. suecica. *(a) Distribution of anti-AtCENH3 (red) and anti-AaCENH3 (green) signals in dividing cells of the newly formed A. thaliana x A. arenosa *A. thaliana x A. arenosa *F1 hybrid, and (b) synthetic A. suecica*A. suecica*.

Supplementary file6 (DOCX 13 KB) List of primers and their sequences

Supplementary file7 (DOCX 16 KB) Localization patterns of subgenome-specific CENH3 variants in nuclei

Supplementary file8 (MP4 29301 KB) A. thaliana*A. thaliana* (red) and A. arenosa *A. arenosa *(green) CENH3-containing chromatin regions intermingle in *A. arenosa x A. thaliana* F1 centromeres (see also Figure 3)

## Data Availability

Enquiries about data availability should be directed to the authors.
