# Supplementary figures and images for: Centromere sequence-independent but biased loading of subgenome-specific CENH3 variants in allopolyploid Arabidopsis suecica
Source: Plant Mol Biol. 2024 Jun 14;114(4):74. doi: 10.1007/s11103-024-01474-5 (PMC11178584; doi:10.1007/s11103-024-01474-5)

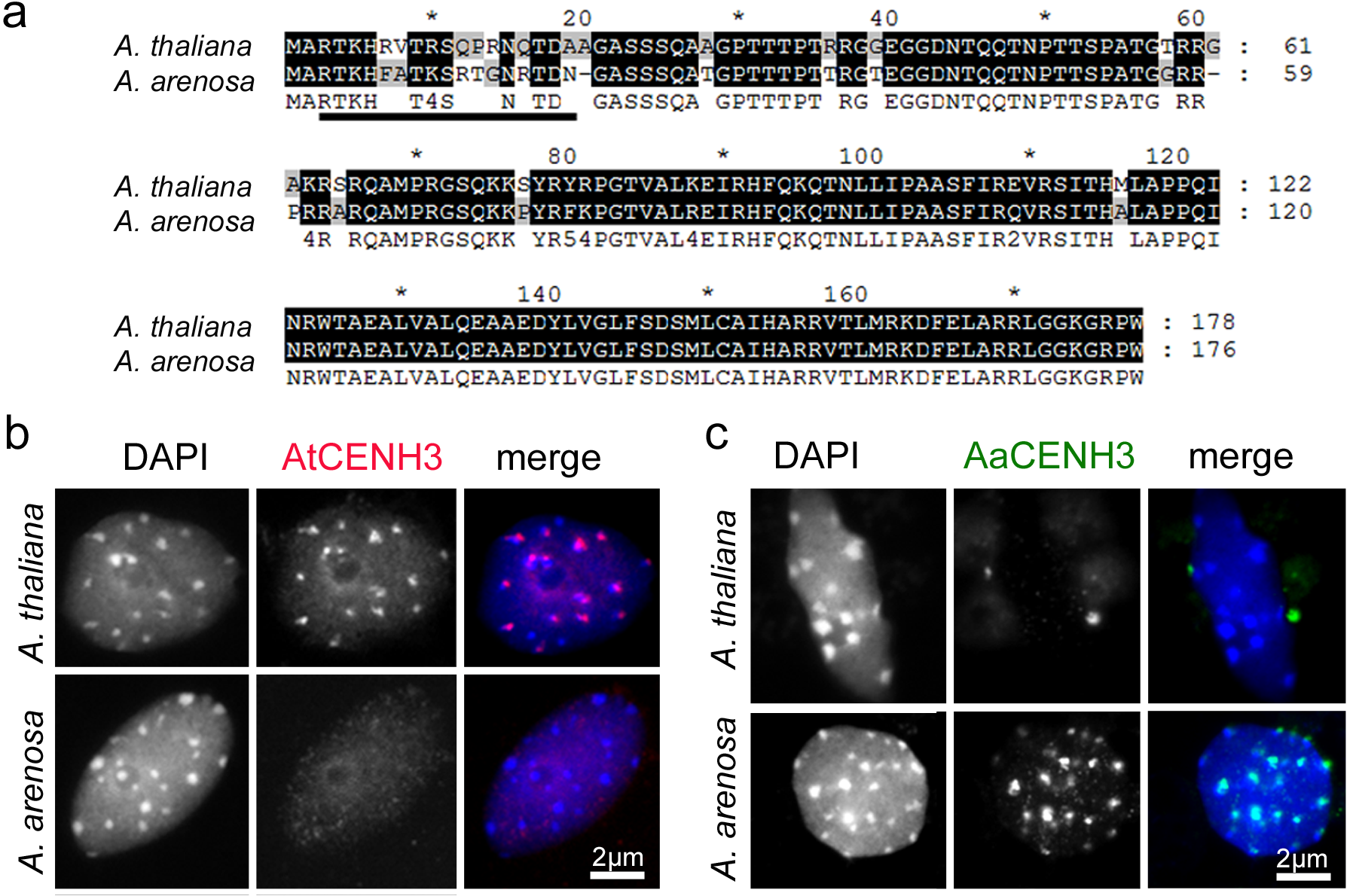

Supplement: Supplementary file 1 — Supplementary file1 (TIF 6281 KB) Generation and characterization of anti-AaCENH3 and anti-AtCENH3 antibodies. (a) Alignment of A. thaliana and A. arenosa A. arenosa CENH3 protein sequences. The polymorphic region (underlined, RTKHFATKSRTGNRTDN) was used to synthesize a peptide for generating AaCENH3-specific antibodies. Immunostaining of A. arenosa and A. thalianaA. thaliana nuclei with (b) anti-AtCENH3 and (c) anti-AaCENH3 antibodies demonstrate species-specific CENH3 recognition [file 11103_2024_1474_MOESM1_ESM.tif]

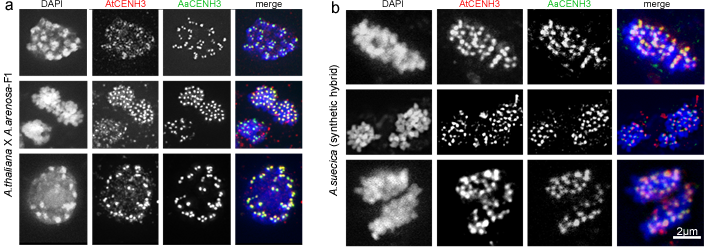

Supplement: Supplementary file 5 — Supplementary file5 (TIF 528 KB) Coexistence of species-specific CENH3 variants in the centromeres of flower bud cells in F1 hybrid and synthetic A. suecica. A. suecica. (a) Distribution of anti-AtCENH3 (red) and anti-AaCENH3 (green) signals in dividing cells of the newly formed A. thaliana x A. arenosa A. thaliana x A. arenosa F1 hybrid, and (b) synthetic A. suecicaA. suecica. [file 11103_2024_1474_MOESM5_ESM.tif]
