# Supplementary material for: Centromere sequence-independent but biased loading of subgenome-specific CENH3 variants in allopolyploid Arabidopsis suecica
Source: Plant Mol Biol. 2024 Jun 14;114(4):74. doi: 10.1007/s11103-024-01474-5 (PMC11178584; doi:10.1007/s11103-024-01474-5)
Supplement: Supplementary file 2 — Supplementary file2 (PPTX 281 KB) Flow cytometric analysis of the progeny of A. thaliana (4x) x A. arenosaA. arenosa (4x) crosses revealed in one plant elimination of all A. arenosa chromosomes. (a) Overlay of independent measurements of A. thalianaA. thaliana (4x) (red) and the putative hybrid plants 6 (grey) indicating its diploid status. (b) Double immunostaining on sorted nuclei of the 2x progeny (plant No. 6) using AtCENH3 (red) and AaCENH3 (green) antibodies. Only AtCENH3-specific and no AaCENH3 signals were found in nuclei, confirming the loss of A. arenosaA. arenosa chromosomes in the F1 plant. Bar represents 10 µm. (c) Overlay histogram of flow cytometric measurements of A. thaliana (4x) (red), A. arenosa A. arenosa (4x) (blue) and all tested putative F1 hybrid plants (grey) except plant 6 indicates severe differences in genome size between individual hybrid plants [file 11103_2024_1474_MOESM2_ESM.pptx]

## Slide 1
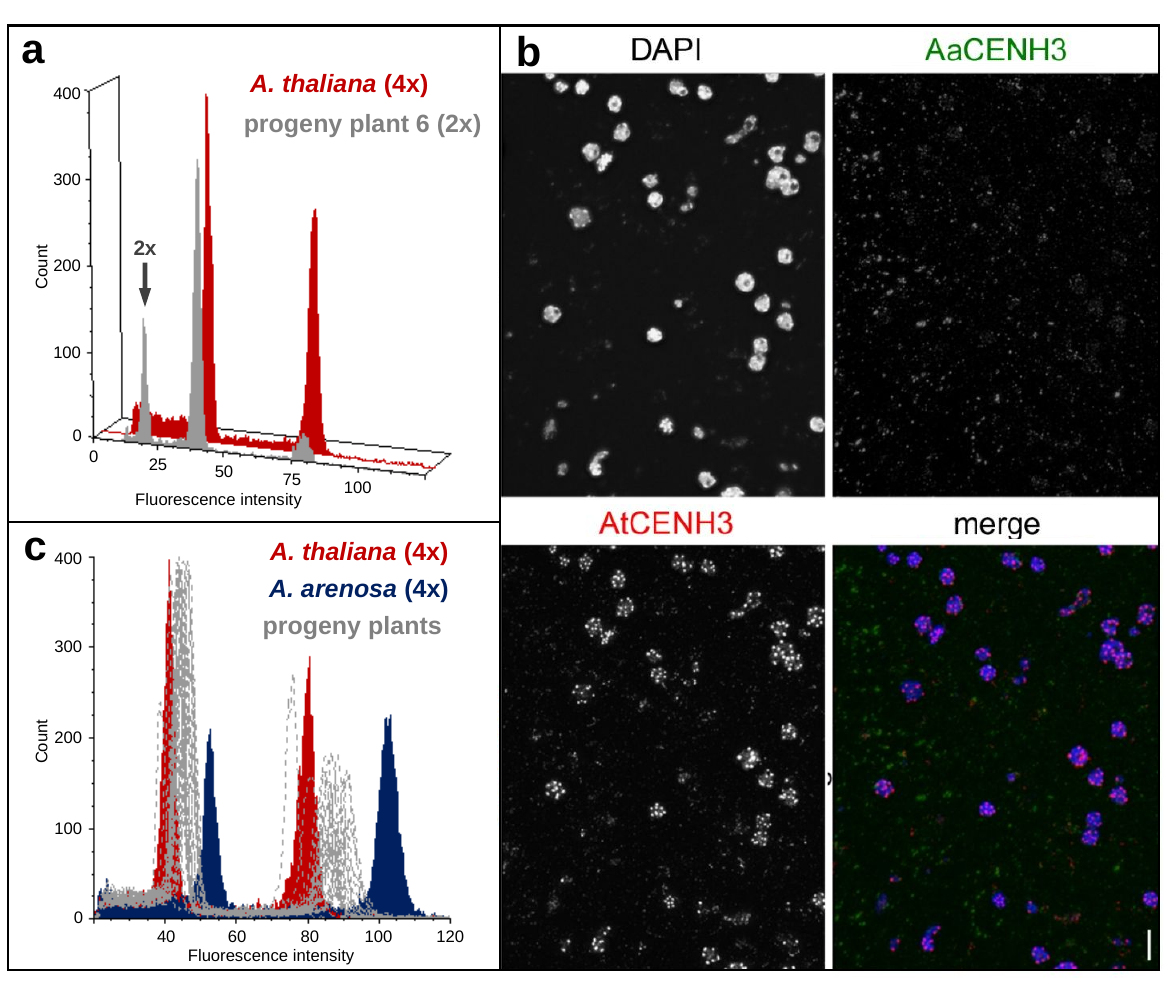

a
b
A. thaliana (4x)
400
progeny plant 6 (2x)
300
2x
200
Count
100
0
0
25
50
75
100
Fluorescence intensity
c
A. thaliana (4x)
400
A. arenosa (4x)
progeny plants
300
200
Count
100
0
40
60
80
100
120
Fluorescence intensity
