# Supplementary material for: Centromere sequence-independent but biased loading of subgenome-specific CENH3 variants in allopolyploid Arabidopsis suecica
Source: Plant Mol Biol. 2024 Jun 14;114(4):74. doi: 10.1007/s11103-024-01474-5 (PMC11178584; doi:10.1007/s11103-024-01474-5)
Supplement: Supplementary file 3 — Supplementary file3 (PPTX 1104 KB) Validation of the hybrid nature of A. thaliana x A. arenosa F1 plants. (a) The DNA content of F1 hybrid plants from crosses between A. thaliana A. thalianaand A. arenosa was determined by flow cytometry. Plants 25 and 27 were quite similar to Col-0, most likely due to failure in cross-pollination and consequently produced by self-pollination. The yellowish area represents the +/- 3% interval around the expected DNA content (0.754 pg/2C) of the hybrid plants based on measurements of both parental genome. Plants inside this interval were considered as euploid F1 hybrids (b) Phenotypes of some analyzed plants by flow cytometry. The images show the haploid plant 6 and the tetraploid A. thaliana(No. 25), along with F1 hybrids of 11, 16, and 24. The upper and lower panels represent side and top views, respectively. Note the differences in phenotypes [file 11103_2024_1474_MOESM3_ESM.pptx]

## Slide 1
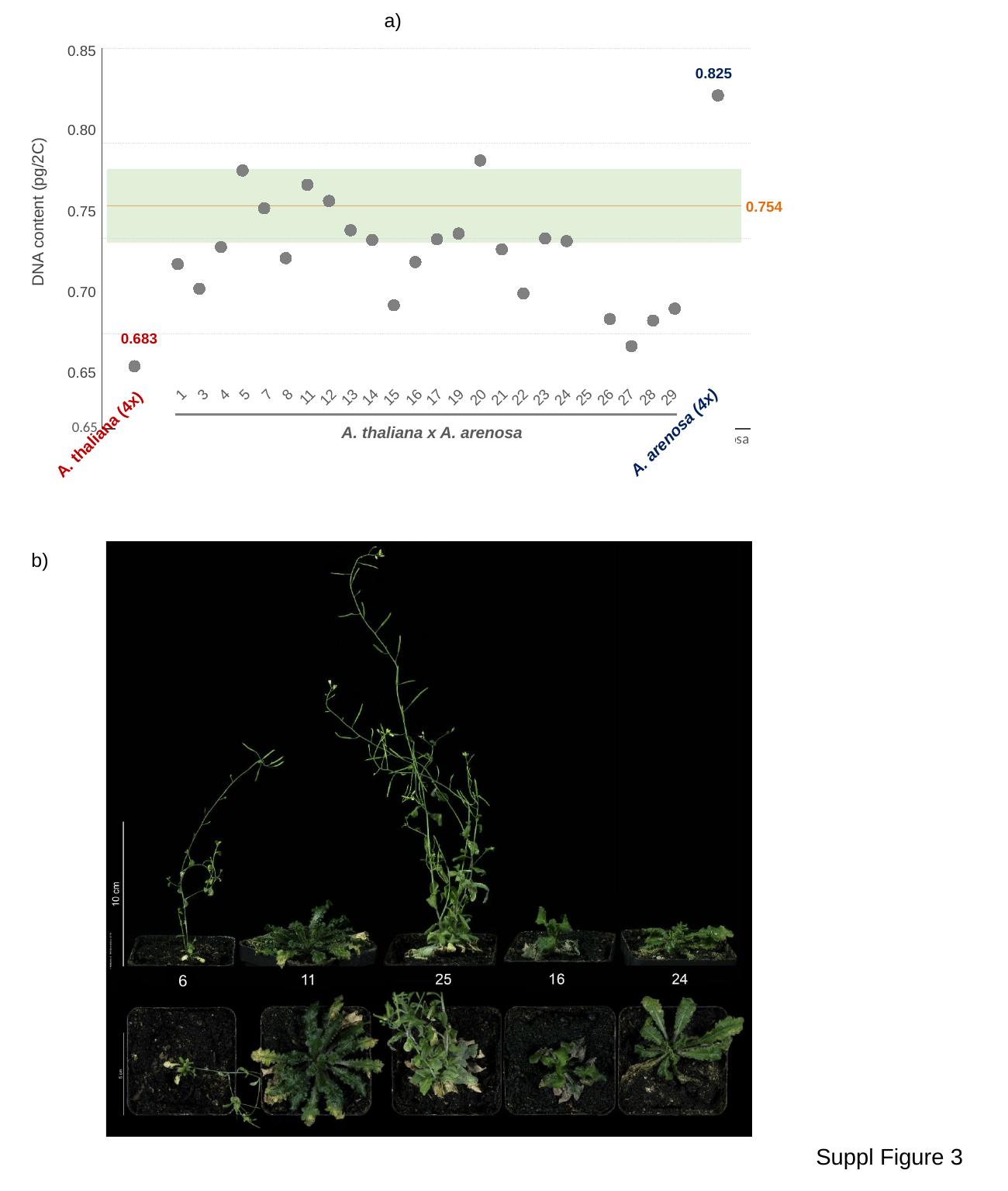

### Chart
| Category | |
|---|---|
| | None |
| N3151 | 0.68289395945881 |
| | None |
| 1 | 0.7365455992389607 |
| 3 | 0.723617212681075 |
| 4 | 0.7455093275172766 |
| 5 | 0.7857829860524805 |
| 7 | 0.7659022026256732 |
| 8 | 0.7397071631220248 |
| 11 | 0.7782632867621143 |
| 12 | 0.7697625326170033 |
| 13 | 0.7543880773786026 |
| 14 | 0.749239657100261 |
| 15 | 0.7149353926141143 |
| 16 | 0.7376355482069776 |
| 17 | 0.7496675200785606 |
| 19 | 0.7526191629194051 |
| 20 | 0.7910641740440708 |
| 21 | 0.7443325526932085 |
| 22 | 0.7210791145726584 |
| 23 | 0.7500533844363836 |
| 24 | 0.7486508740927721 |
| 25 | 0.6618901473796068 |
| 26 | 0.7077331780179109 |
| 27 | 0.693451222257786 |
| 28 | 0.7068788291354663 |
| 29 | 0.71314824035917 |
| | None |
| A. arenosa | 0.8252625464469309 |
0.85
a)
0.825
0.80
0.754
DNA content (pg/2C)
0.75
0.70
0.683
0.65
1
3
4
5
7
8
11
12
13
14
15
16
17
19
20
21
22
23
24
25
26
27
28
29
A. thaliana x A. arenosa
A. thaliana (4x)
A. arenosa (4x)
b)
Suppl Figure 3
6
