# Supplementary material for: Centromere sequence-independent but biased loading of subgenome-specific CENH3 variants in allopolyploid Arabidopsis suecica
Source: Plant Mol Biol. 2024 Jun 14;114(4):74. doi: 10.1007/s11103-024-01474-5 (PMC11178584; doi:10.1007/s11103-024-01474-5)
Supplement: Supplementary file 4 — Supplementary file4 (PPTX 38 KB) The frequency of unbiased and biased CENH3 loaded nuclei A. thaliana x A. arenosaA. thaliana x A. arenosa F1plants, as well as synthetic and natural A. suecica. The percentage of unbiased (i.e., AT/AA and at/aa nuclei) and biased (i.e., AT/aa, at/AA, AT/0, at/0, 0/AA, or 0/aa nuclei) subgenome-specific CENH3 loaded nuclei is represented as a proportion of the total number of nuclei (sum of 2C and 4C) in each genotype [file 11103_2024_1474_MOESM4_ESM.pptx]

## Slide 1
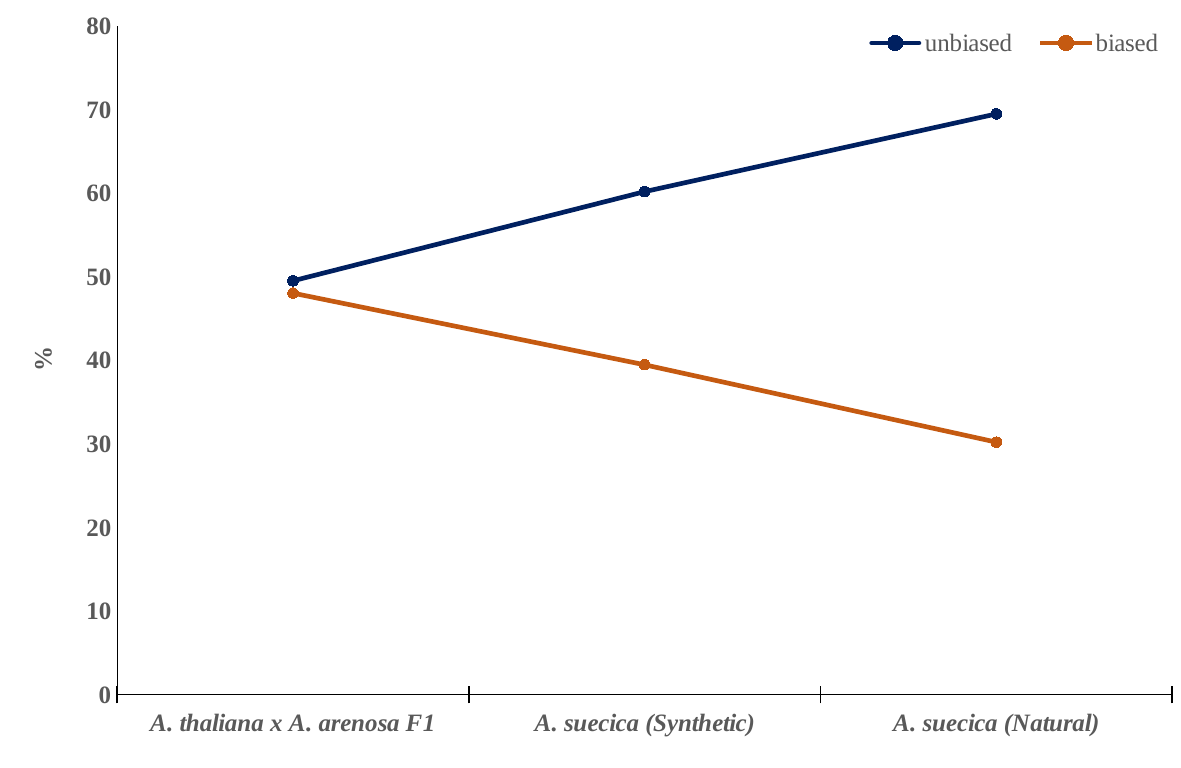

### Chart
| Category | unbiased | biased |
|---|---|---|
| A. thaliana x A. arenosa F1 | 49.524815205913406 | 48.04646251319958 |
| A. suecica (Synthetic) | 60.19736842105263 | 39.473684210526315 |
| A. suecica (Natural) | 69.50146627565982 | 30.205278592375368 |
