# Supplementary material for: Centromere sequence-independent but biased loading of subgenome-specific CENH3 variants in allopolyploid Arabidopsis suecica
Source: Plant Mol Biol. 2024 Jun 14;114(4):74. doi: 10.1007/s11103-024-01474-5 (PMC11178584; doi:10.1007/s11103-024-01474-5)
Supplement: Supplementary file 6 — Supplementary file6 (DOCX 13 KB) List of primers and their sequences [file 11103_2024_1474_MOESM6_ESM.docx]

Supplementary table 1. List of primers and their sequences.

| ELF1-F | AAACCTACATCTCCGGGATCAATT |
| --- | --- |
| ELF1-R | ACAGAAGACTTTCCACTCTCTTTAG |
| Actin 2 (AT3G18780 )-F | TGAGAGATTCAGATGCCCAGAAG |
| Actin 2 (AT3G18780)-R | TGGATTCCAGCAGCTTCCAT |
| AtCEN-F | CATGGTGGTAGCCAAAGTCCATA |
| AtCEN-R | GCTTTGAGAAGCAAGAAGAAGG |
| AaCEN-F | AGCTTCTTATTGCTCTCAACGG |
| AaCEN-R | TTAGAAGCTCCAAAACCGAAAA |
| Aa-CENH3-F | AGGACAGGATCTGGGAATCG |
| Aa-CENH3-R | TGTGAACCTCGTGGCATAG |
| At-CENH3-F | AACCTCGGAATCAAACTGATGCC |
| At-CENH3-R | CGATAAGACTTCTTCTGTGAG |
