# Supplementary material for: Centromere sequence-independent but biased loading of subgenome-specific CENH3 variants in allopolyploid Arabidopsis suecica
Source: Plant Mol Biol. 2024 Jun 14;114(4):74. doi: 10.1007/s11103-024-01474-5 (PMC11178584; doi:10.1007/s11103-024-01474-5)
Supplement: Supplementary file 7 — Supplementary file7 (DOCX 16 KB) Localization patterns of subgenome-specific CENH3 variants in nuclei [file 11103_2024_1474_MOESM7_ESM.docx]

| **Suppl. Table 2 Localization patterns of subgenome-specific CENH3 variants in nuclei** | | | | | | |
| --- | --- | --- | --- | --- | --- | --- |
| Line | C | **anti-AtCENH3** | | **anti-AaCENH3** | | |
|  |  | **AT** | **at** | **AA** | **aa** | **00** |
| *A. thaliana* | 4C | 110 | 41 | 0 | 0 | 3 |
| *A. thaliana x A. arenosa* F1 hybrid | 2C | 206 | 262 | 368 | 118 | 13 |
|  | 4C | 125 | 273 | 333 | 102 | 10 |
| Synthetic *A. suecica*  (N22665) | 2C | 59 | 88 | 150 | 2 | 0 |
|  | 4C | 121 | 30 | 150 | 1 | 1 |
| Natural *A. suecica* (Sue2) | 2C | 94 | 69 | 161 | 3 | 0 |
|  | 4C | 128 | 48 | 163 | 13 | 1 |
| AT: *A. thaliana* CENH3 strong signal , at: *A. thaliana* CENH3 weak signal, AA: *A. arenosa* CENH3 strong signal, aa: *A. arenosa* CENH3 weak signal, 00: undetectable signals | | | | | | |
